# Is Early Monitoring Better? Impact of Early Vancomycin Exposure on Treatment Outcomes and Nephrotoxicity in Patients with Methicillin-Resistant *Staphylococcus aureus* Infections

**DOI:** 10.3390/antibiotics9100672

**Published:** 2020-10-04

**Authors:** Thanawat Chattaweelarp, Dhitiwat Changpradub, Baralee Punyawudho, Sudaluck Thunyaharn, Wichai Santimaleeworagun

**Affiliations:** 1 College of Pharmacotherapy Thailand, Nontaburi 11000, Thailand; thanawat.025@gmail.com; 2 Department of Pharmacy Practice, Faculty of Pharmacy, Payap University, Chiang Mai 50000, Thailand; 3 Division of Infectious Disease, Department of Medicine, Phramongkutklao Hospital, Bangkok 10400, Thailand; dhitiwat@yahoo.com; 4 Department of Pharmaceutical Care Faculty of Pharmacy Chiang Mai University, Chiang Mai 50200, Thailand; baralee.p@cmu.ac.th; 5 Faculty of Medical Technology, Nakhonratchasima College, Nakhon Ratchasima 30000, Thailand; tanmicro@gmail.com; 6 Department of Pharmacy, Faculty of Pharmacy, Silpakorn University, Nakorn Pathom 73000, Thailand; 7 Pharmaceutical Initiative for Resistant Bacteria and Infectious Disease Working Group [PIRBIG], Nakorn Pathom 73000, Thailand

**Keywords:** area under the curve, mortality, MRSA, nephrotoxicity, vancomycin

## Abstract

Optimal early vancomycin target exposure remains controversial. To clarify the therapeutic exposure range, we investigated the association between vancomycin exposure and treatment outcomes or nephrotoxicity in patients with methicillin-resistant *Staphylococcus aureus* (MRSA) infection. This retrospective study reviewed clinical data obtained from 131 patients with MRSA infections between January 2017 and September 2019. Clinical outcomes included treatment failure, 30-day mortality, microbiological failure, and acute kidney injury. We measured serum vancomycin levels after the first dose to 48 h and estimated vancomycin exposure using the Bayesian theorem. The minimum inhibitory concentration (MIC) of antimicrobial agents was determined using the broth microdilution method. Classification and Regression Tree analyses identified day 1 and 2 exposure thresholds associated with an increased risk of failure and nephrotoxicity. Treatment failure (27.9% vs. 33.3%) and 30-day mortality (26.6% vs. 31.74%) were numerically but not significantly reduced in patients with the area under the curve (AUC)_24–48h_/MIC_BMD_ ≥ 698. Patients with AUC_ss_/MIC_BMD_ ≥ 679 exhibited a significantly increased risk of acute kidney injury (27.9% vs. 10.9%, *p* = 0.041). These findings indicate that AUC_ss_/MIC_BMD_ ratios > 600 may cause nephrotoxicity. AUC/MIC_BMD_ at days 1 and 2 do not appear to be significantly associated with particular clinical outcomes, but further studies are needed.

## 1. Introduction

Methicillin-resistant *Staphylococcus aureus* (MRSA) is the most common cause of nosocomial infections. The Asian Network for Surveillance of Resistant Pathogens (ANSORP) study reported that MRSA accounts for 57% of hospital-acquired infections in Thailand [1].

Glycopeptide antibiotic vancomycin remains the treatment of choice for MRSA infections. However, patient conditions can be affected by complex vancomycin pharmacokinetics (PK) and variable serum concentrations. In particular, critically ill patients experience extreme physiological changes [2]. A previous study showed that intensive care unit (ICU) patients had a volume of distribution (Vd) nearly twice as high as that of healthy patients [3]. Moreover, the early phase of sepsis is a hypermetabolic condition that leads to increased renal blood flow and renal elimination of antibiotics, which is a phenomenon termed augmented renal clearance (ARC). ARC is strongly associated with subtherapeutic vancomycin serum concentrations during the first three days of treatment [4]. Thus, standard dosing might lead to insufficient vancomycin exposure and therapeutic failure in these patients [2,3,4,5].

Recently published guidelines for the therapeutic monitoring of vancomycin in patients with serious MRSA infections no longer use a vancomycin trough concentration of 15–20 mg/L. Instead, the area under the curve (AUC)/minimum inhibitory concentration (MIC) ratio is the preferred PK/pharmacodynamics (PD) parameter for predicting vancomycin efficacy. Therefore, in patients with suspected or definitive MRSA infections, an individualized target AUC of 400–600 (assuming a vancomycin MIC_BMD_ of 1 µg/mL or less) is recommended to achieve clinical efficacy and improve nephrotoxicity [6].

A study by Lodise et al. in patients with *S. aureus* bacteremia found that delayed therapy (>45 h) was associated with with higher mortality rates and longer hospitalizations, highlighting the importance of determining the early target concentration of vancomycin [7]. Moreover, vancomycin targeted exposure should be achieved early during therapy, preferably within the first 24 to 48 hrs. As such, using Bayesian-derived AUC monitoring may be practical in these cases, since it does not require steady-state serum vancomycin concentrations to allow for the early assessment of AUC target attainment [6].

A recent study evaluating the association between AUC/MIC values at steady-state (96 h) and 30-day mortality showed that AUC/MIC_BMD_ ratios ≥400 were not associated with lower 30-day all-cause mortality [8]. Two recent studies comparing early vancomycin exposure in patients with MRSA bloodstream infections and treatment failure showed that patients with AUC_0–24h_/MIC_BMD_ ≥ 521 and AUC_24–48h_/MIC_BMD_ ≥ 650 on day two of therapy exhibited reduced treatment failure. However, this result was not significant [9,10]. Moreover, the incidence of vancomycin-associated acute kidney injury (AKI) was correlated with vancomycin exposure at steady-state [11]. Lodise et al. indicated that vancomycin AUC_24–48h_ < 515 was the threshold for minimizing AKI [10]. Thus, both early and steady-state AUC/MIC, values must be re-evaluated in terms of treatment outcomes and nephrotoxicity [12].

However, most previous studies have measured serum vancomycin concentrations at steady-state (before the fourth dose of vancomycin). Therefore, these results may not reflect the early phase of sepsis patients. Moreover, limited published studies have used the first administration of vancomycin to estimate exposure and MRSA treatment clinical outcomes. In this study, the objective was to evaluate the impact of vancomycin exposure (AUC/MIC) during the early phase and at a steady-state on treatment outcomes and nephrotoxicity in patients with MRSA infections.

## 2. Results

From January 2017 to September 2019, we obtained results from 131 MRSA infection patients with 315 measured vancomycin concentrations. Of the 131 patients with MRSA infections, 88 (67.2%) were men, the mean age was 70.1 years, and there were 82 (62.6%) critically ill patients. All patients received intravenous vancomycin for more than 48 h. Most patients were diagnosed with pneumonia and bacteremia. The MIC range, MIC50, and MIC90 for vancomycin against the studied MRSA isolates were 0.5–2.0 µg/mL, 1 µg/mL, and 1 µg/mL, respectively (Table 1). Table 3 shows the vancomycin exposures (AUC/MIC_BMD_) and clinical outcomes.

### 2.1. Efficacy

Of the 131 patients, 40 (30.5%) patients experienced vancomycin treatment failure, 38 (29%) were dead within 30 days of the index culture, and 6 (4.6%) had microbiological failure despite seven days of vancomycin therapy (Table 2).

According to Classification and Regression Tree (CART) analysis, there were no significant differences in 30-day mortality or microbiological failure between patients with high (≥) or low (<) vancomycin exposures for AUC_0–24h_/MIC_BMD_ 626, AUC_24–48h_/MIC_BMD_ 698, and AUC_ss_/MIC_BMD_ 679. Treatment failure did not differ between high and low vancomycin exposures for AUC_0-24h_/MIC_BMD_ 626 (32.8% vs. 28.1%, *p* = 0.558) and AUC_24–48h_/MIC_BMD_ 698 (33.3% vs. 27.9%, *p* = 0.503), respectively (Table 3). According to the exploratory analyses from previous studies, an AUC_0–24h_/MIC_BMD_ of 521 and AUC_24–48h_/MIC_BMD_ of 650 were not associated with treatment failure.

### 2.2. Nephrotoxicity

**Figure 1:**
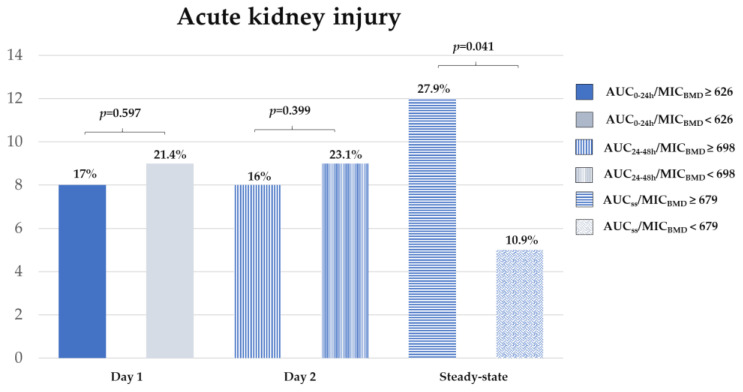
Relationship between Classification and Regression Tree (CART) analysis-derived area under the curve/MIC exposure variables and acute kidney injury. Abbreviations: AUC, area under the curve; BMD, broth microdilution; CART, Classification and Regression Tree; MIC, minimum inhibitory concentration, SS, steady-state.

**Figure 2:**
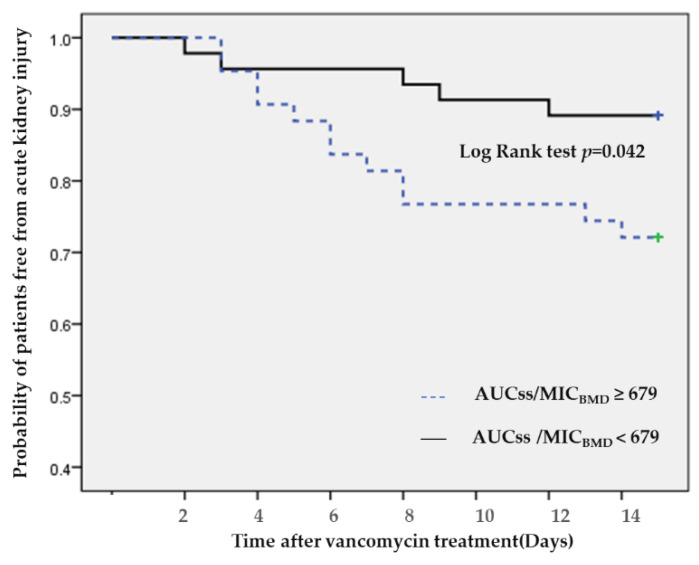
Kaplan–Meier survival analysis of acute kidney injury-free events between higher and lower AUC/MIC_BMD_ breakpoints (AUC_ss_/MIC_BMD_ 679). Abbreviations: AUC, area under the curve; BMD, broth microdilution; MIC, minimum inhibitory concentration; AKI, acute kidney injury, SS, steady-state.

Eighty nine patients had a baseline serum creatinine level <2.0 mg/dL. According to CART analysis-derived vancomycin exposure values, an AUC_ss_/MIC_BMD_ of 679 was the breakpoint for AKI. The number of patients with AKI was 17, and was significantly higher in the high vancomycin exposure groups (AUC_ss_/MIC_BMD_ 679 (27.9% vs. 10.9%, *p* = 0.041)) (Figure 1). Kaplan–Meier survival analysis of the time to AKI demonstrated a significantly increased risk of AKI in the high AUC group (>670) (log-rank test *p* = 0.042) (Figure 2).

## 3. Discussion

This study evaluated vancomycin exposure during the first dose of its administration, and the resultant clinical outcomes, in patients with MRSA infections. These results were obtained using broth microdilution methods to measure MICs (the gold standard method for vancomycin susceptibility determination) [9,10,13]. We estimated the AUC values using individual vancomycin levels. We used vancomycin concentrations after the first dose to calculate the AUC values that reflected vancomycin PK parameters in patients during the first 24 h. In contrast, previous studies often determined vancomycin concentrations at steady-state, which may be in the transition to the recovery phase of sepsis.

The current guidelines, provided by the American Society for Health System Pharmacists (ASHP)/Infectious Diseases Society of America (IDSA)/the Pediatric Infectious Diseases Society (PIDS/the Society of Infectious Diseases Pharmacists (SIDP), no longer recommend trough concentrations of 15–20 mg/L. AUC-based dosing is preferable because AUC_24_/MICss is the most accurate PK/PD parameter. In patients with suspected or definitive serious MRSA infections, an individualized target of an AUC/MIC_BMD_ ratio between 400 and 600 (assuming a vancomycin MIC_BMD_ of 1 µg/mL) is recommended to achieve clinical efficacy and improve patient safety [6]. This target value came from studies on patients with MRSA bacteremia; most studies estimated PK/PD targets using formulas [8,14,15,16]. This study used Bayesian software to estimate AUC values, which provides an advantage in critically ill patients since vancomycin concentrations could be collected within the first 24 to 48 h, rather than at steady-state conditions (after the third or fourth dose) [6].

Moreover, the current guidelines recommend Bayesian-derived AUC monitoring based on Bayes’s theorem. Bayesian dose optimization software uses a well-developed vancomycin population PK model as the Bayesian prior, together with the individual patient’s measured drug concentrations in the data file, to calculate a Bayesian posterior parameter value distribution for that patient. Pea et al. assessed therapeutic drug monitoring (TDM) using a Bayesian approach to determine vancomycin dosages compare with a nomogram in critically ill patients. The study suggested that a TDM-guided Bayesian-based approach should be considered an invaluable tool for clinicians to appropriately monitor real-time vancomycin therapy in critically ill patients [17]. In this study, peak and trough vancomycin concentrations were measured in over half the patients for whom the previous study showed peak-trough based vancomycin TDM improved the therapeutic cure rate [18].

Using this validated Bayesian method to estimate the daily AUC in a single-center, retrospective study of patients with MRSA bloodstream infections, Lodise et al. found that failure outcomes were maximized when day 1 and day 2 AUC/MIC_BMD_ ratios exceeded 521 and 650, respectively [9]. A multicenter, observational prospective study was performed to evaluate the relationship between prespecified day 2 AUC/MIC ratios (AUC/MIC_BMD_ of ≥ 650) and outcomes in adult patients with MRSA bacteremia. The study, performed by Ho et al. in MRSA bacteremia patients, showed that treatment outcomes were improved by adjusting the dose to achieve an AUC_24_/MIC_BMD_ of > 400 based on individual vancomycin clearances and the vancomycin MIC of the infection-causing strain [19].

In our study, higher vancomycin exposures were not associated with treatment failure, 30-day mortality, or microbiological failure. In contrast, AKI was associated with higher AUC_ss_/MIC_BMD_ values ≥679. In the previous multivariate analyses, treatment failure rates were not significantly different between the pre-specified day 2 AUC/MIC groups [10], as our study found that treatment failure was not significantly associated with higher vancomycin exposures on day 1 (AUC/MIC_BMD_ of ≥626) and day 2 (AUC/MIC_BMD_ of ≥698). Approximately 32% of patients in our study presented with hemodialysis and renal failure in critical condition, including continuous renal replacement therapy (CRRT). Even though the AUC/MIC values were higher than in previous studies, patient condition trends in the higher 30-mortality rate from a study in end-stage renal disease (ESRD) patients in an ICU setting were about 20–40%, compared with those without renal failure [20,21]. Based on our findings (data not shown in the results part), the patients with ESRD had a significantly higher mortality rate than the non-ESRD group (42.86% vs. 22.47%).

Not surprisingly, the CART-derived exposure AUC_ss_/MIC_BMD_ values showed that the rates of AKI were significantly higher in patients in the high vancomycin exposure groups (AUC_ss_/MIC_BMD_ 679 (27.9% vs. 10.9%, *p* = 0.041)]). The AUC breakpoint associated with an increased risk of AKI in this study was notably consistent with previous reports [10,11,22,23,24,25]. However, in our analysis, there was no correlation between AUC_0–24h_/MIC_BMD_ (626) and AUC_24–48h_/MIC_BMD_ (698) with nephrotoxicity, unlike in a previous study [22]. The data from a previous meta-analysis [26,27] were similar to the updated guidelines using AUC-guided dosing for reduced nephrotoxicity at values lower than 650. Notably, the higher AUC value cited (above 600 mg h/L) provides a new index that incorporates both efficacy and AKI, and is within the recommended AUC range of 400 to 600 mg h/L (assuming an MIC of 1 mg/L) [6,28].

This study has several limitations. First, our study used Bayesian software that uses a one-compartment model as a prior PK parameter, which may not reflect that our study included about 60% critically ill patients. However, previous population PK studies in critically ill or sepsis/septic shock patients used both a one- and two-compartment model. Second, the sample size in our study was small. Thus, a larger MRSA population may show significant differences in treatment outcomes. Third, vancomycin measurement assays in this study had been reported to impair reaction kinetics leading to the incorrect results, especially below the measuring range (<4.0 μg/mL). However, in this study, patients in the higher group of vancomycin exposure tended to show better treatment outcomes than those in the lower exposure groups.

## 4. Materials and Methods

### 4.1. Study Design

This retrospective study was conducted between January 2017 and September 2019 at Phramongkutklao Hospital, a 1200-bed university hospital in Bangkok, Thailand. Patients who were admitted with an MRSA infection, treated with intravenous vancomycin infusion, and had serum vancomycin concentration data were included.

The ethics review committee of the Royal Thai Army Medical Department, Bangkok, Thailand (approval no. Q0007h/63_Exp) approved this study. It was performed following the Declaration of Helsinki. Data were collected after obtaining ethical approval and permission from the Director of the Phramongkutklao Hospital.

Patient data were collected and analyzed anonymously and confidentially. Identifying information of participants was not collected, and only the researcher could access the data. The ethics review committee did not require patient informed consent for using serum vancomycin levels, retrospective chart review studies, and confidential and anonymized data.

### 4.2. Microbiological Data

We determined the MIC of the non-repeated clinical isolates. The MIC of the antimicrobial agents was determined using automated susceptibility testing (Thermo Scientific™ Sensititre™ ARIS™ 2X Instrument) based on the broth microdilution method. The MIC value of the antimicrobials in each strain was denoted as susceptible, intermediate, or resistant using the Clinical and Laboratory Standards Institute (CLSI) breakpoint [29].

### 4.3. Vancomycin Assay

Serum vancomycin concentrations were determined using a fluorescence immunoassay (VANC3, Cobas, Roche Diagnostics, Indianapolis, USA). The limit of detection of this assay for vancomycin was 4 µg/mL. The coefficient of variation for this assay was <10%.

### 4.4. Treatment Data and Outcomes

Patient data were obtained from electronic medical records. Information collected included demographic data, infection dates, specimens for microbial culture, types of infections, the severity of illness, antimicrobial susceptibility, treatment duration, and treatment outcomes.

Serum vancomycin concentrations were collected at 1–2 h after the end of the infusion of the loading dose, and 30 min before the second dose or 15 min before the fourth vancomycin dose. The 24 h AUCs for vancomycin were estimated using Bayesian dose optimizing software by Precise PK (San Diego, CA, USA).

The primary outcome measure was treatment failure, defined as any of the following: (1) death within 30 days after treatment (30-day mortality), (2) microbiological failure (specimen culture growing MRSA obtained seven days after the initiation of therapy). Secondary outcome measures included the occurrence of AKI among patients with a baseline serum creatinine <2 mg/dL. AKI was based on post-baseline serum creatinine levels ≥1.5×baseline serum creatinine or an increase >0.5 mg/dL, and was assessed from the initiation of vancomycin to 48 h after initiation [30]. Clinical outcomes were indicated with an AUC_0–24h_/MIC_BMD_ of 521 and AUC_24–48h_/MIC_BMD_ of 650, from previous studies [9,10].

### 4.5. Statistical Analysis

We reported the descriptive statistics for the clinical characteristics and the MICs at the 50th (MIC50) and 90th percentile (MIC90). CART analysis was used to determine the AUC/MIC cut-off value for vancomycin treatment outcomes (included treatment failure, 30-day mortality, microbiological failure, and nephrotoxicity). The Chi-square test was used to compare categorical variables between groups. The survival analysis of AKI used Kaplan–Meier estimates. Data were analyzed in Statistical Package for the Social Sciences (SPSS) version 27.0 (IBM Corp., Armonk, NY, USA), and a *p*-value <0.05 was considered statistically significant.

## 5. Conclusions

Our findings suggest that AUC/MIC_BMD_ values on days 1 and 2 were not associated with treatment outcomes for patients with MRSA infections. However, this study confirmed that nephrotoxicity increased in patients with a high AUC_ss_/MIC_BMD_ of 679. AUC/MIC_BMD_ values of 400–600 remain the best target window to achieve favorable outcomes and minimize the likelihood of AKI, whereas the early threshold of vancomycin exposures remains unclear.

## Figures and Tables

**Table 1:** Characteristics and vancomycin exposure variables.

Characteristics	Values
Gender, male, n (%)	88 (67.2)
Age, years, mean (SD)	70.1 (15.8)
Weight, kg, mean (SD)	61.5 (14.9)
Creatinine clearance, mL/min, mean (SD)	30.9 (29.5)
Critically ill*, n (%)	82 (62.6)
Indication, n (%)	
Pneumonia	56 (42.7)
Bacteremia	39 (29.8)
Urinary tract infection	5 (3.8)
Skin and soft tissue infections	18 (13.7)
Catheter-related bloodstream infection	3 (2.3)
Central nervous system infection	2 (1.5)
Septic joint infection	4 (3.1)
Others	4 (3.1)
Microbiological phenotypes	
MIC_BMD_ range (µg/mL)	0.5–2
0.5, n (%)	39 (29.8)
1, n (%)	84 (64.1)
2, n (%)	8 (6.1)
MIC_BMD_50/90	1/1
Vancomycin exposure variables, mean (SD)	
AUC_0–24h_	561.1 (182.9)
AUC_0–24h_/MIC_BMD_	706.6 (365.3)
AUC_24–48h_	646.7 (311.2)
AUC_24–48h_/MIC_BMD_	802.7 (492.3)
AUC_ss_	666.8 (360.1)
AUC_ss_/MIC_BMD_	838.9 (446.6)

* Critically ill was defined as patients with an APACHE II score ≥15 and/or SOFA score ≥2 points. Abbreviations: APACHE, acute physiology and chronic health evaluation; SOFA, sequential organ failure assessment; AUC, area under the curve; BMD, broth microdilution; MIC, minimum inhibitory concentration; SS, steady-state; MIC50/90, a minimum concentration that inhibits 50% and 90% of bacterial isolates; SD, standard deviation.

**Table 2:** Clinical outcomes.

Outcomes	n (%)
Treatment failure	40 (30.5)
30-day mortality	38 (29)
Microbiological failure	6 (4.6)
Acute kidney injury	17 (19.1)

**Table 3:** Association between the Classification and Regression Tree (CART) analysis-derived vancomycin area under the curve exposure and treatment failure, 30-day mortality, and microbiological failure.

Vancomycin Exposure		*n*	Treatment Failure		30-Day Mortality		Microbiological Failure	
			n (%)	*p*-value	n (%)	*p*-value	n (%)	*p*-value
AUC_0__–__24h_/MIC_BMD_	≥626	67	22 (32.8)	0.558	20 (29.9)	0.828	3 (4.5)	0.954
	<626	64	18 (28.1)		18 (28.1)		3 (4.7)	
AUC_24__–__48h_/MIC_BMD_	≥698	68	19 (27.9)	0.503	18 (26.5)	0.506	3 (4.4)	0.924
	<698	63	21 (33.3)		21 (31.7)		3 (4.8)	
AUC_ss_ /MIC_BMD_	≥679	65	20 (30.8)	0.954	18 (27.7)	0.742	3 (4.6)	0.985
	<679	66	20 (30.3)		20 (30.3)		3 (4.5)	

Abbreviations: AUC, area under the curve; BMD, broth microdilution; MIC, minimum inhibitory concentration; SS, steady-state.
